# Bivalirudin in Combination with Heparin to Control Mesenchymal Cell Procoagulant Activity

**DOI:** 10.1371/journal.pone.0042819

**Published:** 2012-08-10

**Authors:** Xavier Stephenne, Emanuele Nicastro, Stephane Eeckhoudt, Cedric Hermans, Omar Nyabi, Catherine Lombard, Mustapha Najimi, Etienne Sokal

**Affiliations:** 1 Université Catholique de Louvain, Institut de Recherche Expérimentale et Clinique, Unité de Recherche PEDI, Brussels, Belgium; 2 Cliniques Universitaires Saint Luc, Service de Gastroentérologie et Hépatologie Pédiatrique, Brussels, Belgium; 3 Cliniques Universitaires Saint Luc, Laboratoire d’Hématologie–Hémostase, Brussels, Belgium; 4 Cliniques Universitaires Saint Luc, Service d’Hématologie, Unité d’Hémostase-Thrombose, Brussels, Belgium; Children’s Hospital Boston, United States of America

## Abstract

Islet and hepatocyte transplantation are associated with tissue factor-dependent activation of coagulation which elicits instant blood mediated inflammatory reaction, thereby contributing to a low rate of engraftment. The aim of this study was i) to evaluate the procoagulant activity of human adult liver-derived mesenchymal progenitor cells (hALPCs), ii) to compare it to other mesenchymal cells of extra-hepatic (bone marrow mesenchymal stem cells and skin fibroblasts) or liver origin (liver myofibroblasts), and iii) to determine the ways this activity could be modulated. Using a whole blood coagulation test (thromboelastometry), we demonstrated that all analyzed cell types exhibit procoagulant activity. The hALPCs pronounced procoagulant activity was associated with an increased tissue factor and a decreased tissue factor pathway inhibitor expression as compared with hepatocytes. At therapeutic doses, the procoagulant effect of hALPCs was inhibited by neither antithrombin activators nor direct factor Xa inhibitor or direct thrombin inhibitors individually. However, concomitant administration of an antithrombin activator or direct factor Xa inhibitor and direct thrombin inhibitor proved to be a particularly effective combination for controlling the procoagulant effects of hALPCs both *in vitro* and *in vivo*. The results suggest that this dual antithrombotic therapy should also improve the efficacy of cell transplantation in humans.

## Introduction

Adult mesenchymal stem/progenitor cells are currently under evaluation in several clinical trials. A main concern for clinicians and health authorities is the risk of therapy-induced thrombosis, which has been reported in several patients [1].

We previously showed that hepatocyte transplantation results in clinical benefits to patients with inborn errors of metabolism, for whom this technique may be proposed as an alternative or at least, a bridge to orthotopic liver transplantation [2]–[7]. However, it was important to increase the degree of cell engraftment to improve the clinical outcome of this procedure. One limitation is the finding that isolated hepatocytes exhibit procoagulant activity (PCA), which was found to be linked to tissue factor (TF) expression. This *in vitro* observation has been found to translate clinically in modifications in coagulation parameters and D-dimer levels in recipients of liver cell transplantation, which is suggestive of infraclinical micro-thrombotic events [8].The TF dependent PCA of hepatocytes has more recently been confirmed by an independent team which reported that all the parameters of the instant blood mediated inflammatory reaction (IBMIR) were documented in a tubing loop model, whole blood coagulation model mimicking blood circulation [9]. Furthermore, the activation of the coagulation cascade was previously shown to be associated with negative clinical outcome following pancreatic islet transplantation. PCA not only led to thrombotic events, but also elicited inflammatory reactions involving the up-regulation of adhesion molecule expression and chemokine production, two critical pathways affecting graft success rate [10]–[13].

### Targeting Cell PCA is thus Essential to Improve Safety and Success of Cell Transplantation

We previously isolated human adult liver-derived mesenchymal progenitor cells (hALPCs) following human liver enzymatic digestion [14]. These cells were able to proliferate, but also differentiate into hepatocyte-like cells both *in vitro* and *in vivo*. Therefore, hALPCs represent an attractive cell source for the treatment of liver-based metabolic diseases.

In this context, the aim of the present study was to determine whether hALPCs display PCA and if hALPCs PCA is related to TF expression. In addition, we compared PCA that of other mesenchymal cells like bone marrow- mesenchymal stem cells, skin fibroblats and liver myofibroblasts. Finally, we investigated how this activity could be modulated by evaluating different antithrombotic strategies targeting clotting factors IIa and Xa.

## Results

### Procoagulant Activity of hALPCs

**Figure 1.-hALPCs:**
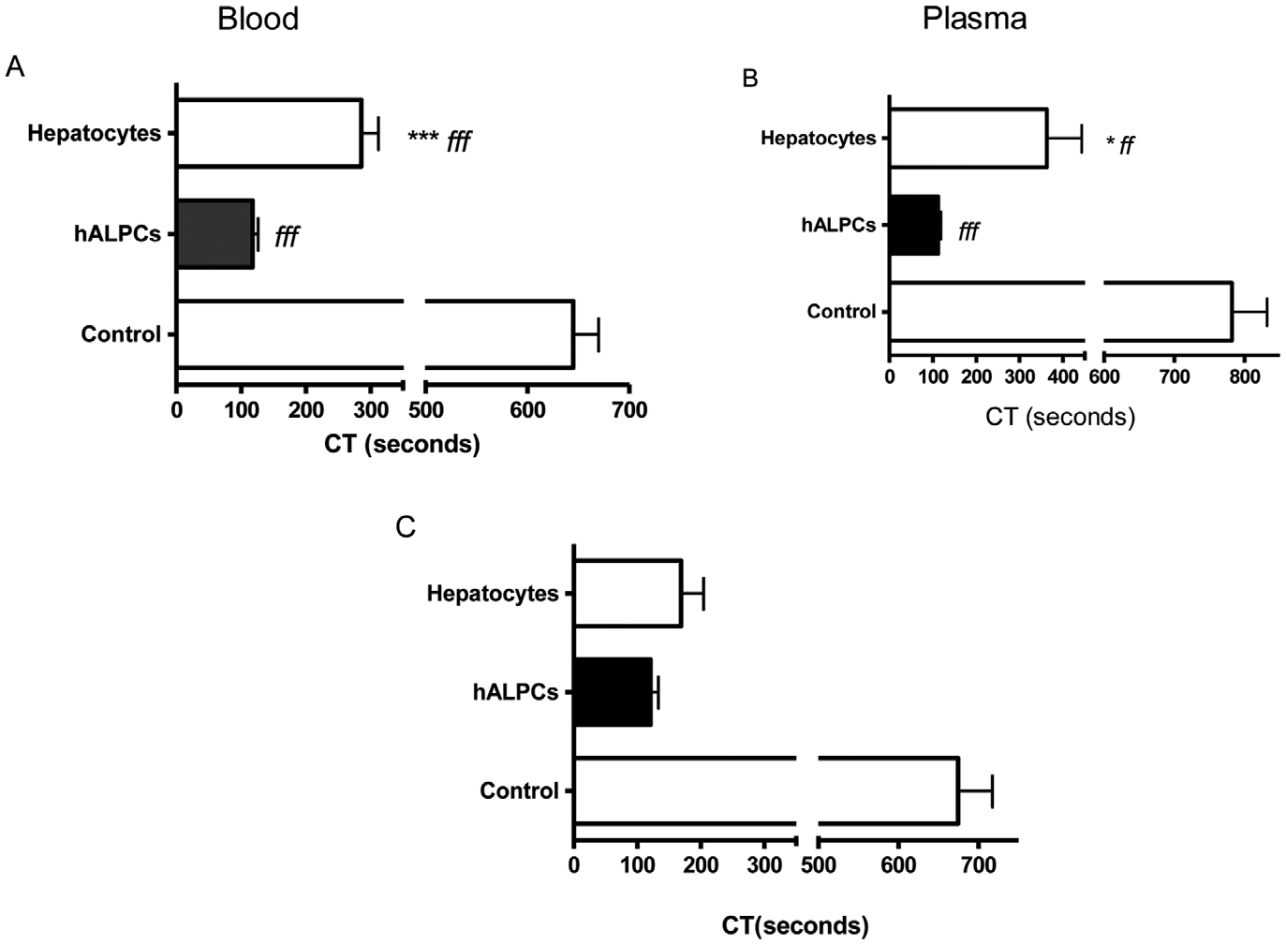
hALPCs PCA in ROTEM. Figure 1A-hALPCs PCA in ROTEM (Blood). Clotting time (CT) assayed by ROTEM after recalcification, with added tissue factor (ExTem 20 µL) of citrated whole blood (300 µl) in presence or not of cells suspended in human albumin 5%. No coagulation was induced in the absence of recalcification. Hepatocytes (white), human adult liver progenitor cells (hALPCs) (black), control (albumin) (grey). * as compared to hALPCs *f* as compared to control hALPCs *vs.* hepatocytes *vs.* control. ***p<0.001 (Kruskal-Wallis test). Figure 1B-hALPCs PCA in ROTEM (Plasma). Clotting time (CT) assayed by ROTEM after recalcification, with added tissue factor (ExTem 20 µL) of plasma (300 µl) obtained from blood incubated in presence or not of cells suspended in human albumin 5%. Hepatocytes (white), human adult liver progenitor cells (hALPCs) (black), control (albumin) (grey). * as compared to hALPCs *f* as compared to control hALPCs *vs.* hepatocytes *vs.* control. ***p<0.001 (Kruskal-Wallis test). Figure 1C-hALPCS PCA in ROTEM (no TF addition). Clotting time (CT) essayed by ROTEM after recalcification, without added Tissue Factor (ExTem 20 µL), of citrated whole blood (300 µl) in presence or not of cells suspended in human albumin 5%. No coagulation is induced if absence of recalcification. Hepatocytes (white), hALPCs (black).

The PCA of hALPCs was determined using thromboelastometry in human whole blood and plasma. The clotting time (CT) of hALPCs was shorter than that of hepatocytes when evaluated using the thromboelastogram in both blood and plasma (116.5±33.7 sec [n = 17] *vs.* 285.8±87.0 sec [n = 11] for blood, p<0.001) (112.6±18.4 sec [n = 9] *vs.* 363.0±180.1 sec [n = 5] for plasma, p<0.05) (Figures 1A and 1B). The control CT without the addition of cells was measured at 644.8±108.8 sec (n = 19) in blood and 781.9±150.5 (n = 9) in plasma. Comparable PCA of hALPCs was observed without adding extrinsic TF (Figure 1C). No PCA was obtained when hALPC culture supernatant was placed in the thromboelastogram instead of cells (Figure S1).

The PCA of hALPCs was also evaluated in the tubing loop model. A decrease in platelet count and increase in D-dimer levels were observed after the incubation of hALPCs with whole blood, with a platelet count decreasing from 295 000/µl to 109 000/µl (Experiment 1) and from 310 000/µl to 134 000/µl (Experiment 2), while D-dimer levels increased from 100 ng/ml to 700 ng/ml (Experiment 1) and from 95 ng/ml to 740 ng/ml (Experiment 2).

### Procoagulant Activity of Mesenchymal Cells

**Figure 2:**
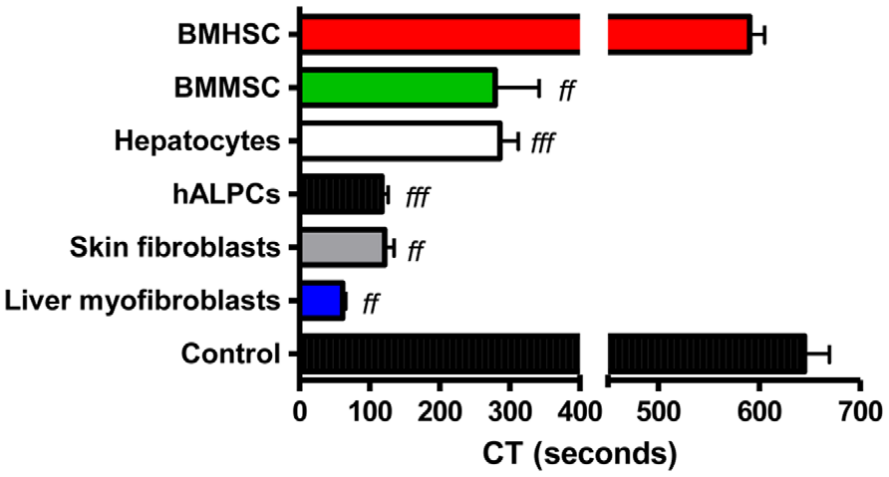
Mesenchymal cells PCA. Clotting time (CT) assayed by ROTEM after recalcification, with added tissue factor (ExTem 20 µL) of citrated whole blood (300 µl) with human adult liver progenitor cells (hALPCs), hepatocytes, skin fibroblasts, bone marrow mesenchymal stem cells (BMMSC), bone marrow haematopoietic stem cells (BMHSC), or liver myofibroblasts suspended in human albumin 5% *f* as compared to control.

The PCA of bone marrow mesenchymal stem cells (279.3±108.3 sec [n = 3], p<0.01 as compared to control), skin fibroblasts (121.8±26.53 sec [n = 3], p<0.01 as compared to control), and liver myofibroblasts (61.7±7.6 sec [n = 3], p<0.01 as compared to control) was evaluated using thromboelastometry on human whole blood. Bone marrow haematopoietic stem cells were used as a control for non-procoagulant cells (590.7±25.3 sec [n = 3]) (Figure 2).

### Modulation of Procoagulant Activity of hALPCs

**Figure 3:**
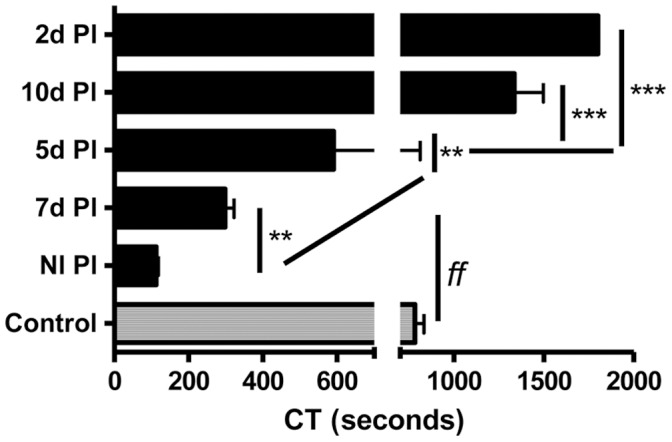
HALPCs PCA in deficient plasma. Clotting time (CT) assayed by ROTEM after recalcification, with added tissue factor (ExTem 20 µL) of plasma (300 µl) deficient in coagulation factors VII, V, X, and II (7d Pl, 5d Pl, 10d Pl, and 2d Pl, respectively) in presence of cells suspended in human albumin 5%. Human adult liver progenitor cells (hALPCs) (black), control (albumin) (grey). * as compared to normal plasma *f* as compared to control.

**Figure 4:**
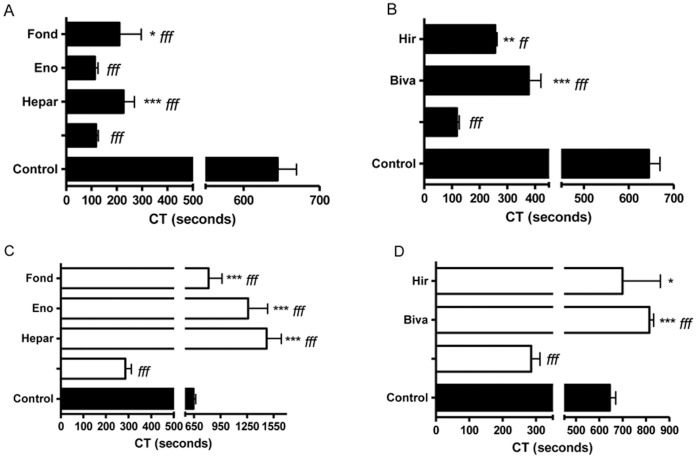
Modulation of hALPCs PCA by anticoagulants. Figure 4A Clotting time (CT) assayed by ROTEM after recalcification, with added tissue factor (ExTem 20 µL) of citrated whole blood (300 µl) in presence or not of human adult liver progenitor cells (hALPCs) suspended in human albumin 5% with heparin (Hepar). In contrast, enoxaparin (Eno) or Fondaparinux (Fond) was extemporaneously added to blood in contact with cells suspended in albumin. hALPCs (black), Control (albumin) (grey). * as compared to hALPCs *f* as compared to control Figure 4B Clotting time (CT) assayed by ROTEM after recalcification, with added tissue factor (ExTem 20 µL) of citrated whole blood (300 µl) in presence or not of human adult liver progenitor cells (hALPCs) suspended in human albumin 5%. Bivalirudin (Biva) or Hirudin (Hir) was extemporaneously added to blood. hALPCs (black), Control (albumin) (grey). * as compared to hALPCs *f* as compared to control Figure 4C Clotting time (CT) assayed by ROTEM after recalcification, with added tissue factor (ExTem 20 µL) of citrated whole blood (300 µl) in presence or not of hepatocytes suspended in human albumin 5% with heparin (Hepar). In contrast, enoxaparin (Eno) or Fondaparinux (Fond) was extemporaneously added to blood in contact with cells suspended in albumin. Hepatocytes (white), Control (albumin) (grey). * as compared to hepatocytes *f* as compared to control Figure 4D Clotting time (CT) assayed by ROTEM after recalcification, with added tissue factor (ExTem 20 µL) of citrated whole blood (300 µl) with or without hepatocytes suspended in human albumin 5%. Bivalirudin (Biva) or Hirudin (Hir) was extemporaneously added to blood. Hepatocytes (white), Control (albumin) (grey). * as compared to hepatocytes *f* as compared to control.

The PCA of hALPCs was first analysed in coagulation factor-deficient plasma. When using factor VII deficient plasma, the physiological cofactor of TF, the PCA of hALPCs was only partially decreased (298.3±42.3 sec [n = 3], p<0.01) compared with the PCA in non-deficient plasma (Figure 3). The PCA of hALPCs was not observed in factor II (thrombin) or X deficient plasma, nor in factor V deficient plasma (Figure 3). The PCA of hALPCs was not fully inhibited by unfractionated heparin (225.8±149.8 sec [n = 15], p<0.001 as compared to control), low molecular weight heparin (112.3±22.5 sec [n = 3], p<0.001 as compared to control), or fondaparinux (209.7±149.7 sec [n = 3], p<0.001 as compared to control) (Figure 4A), even when using dosage increases up to five times (Figure S2). No coagulation was observed when heparin was used in the absence of cells.

The direct thrombin inhibitor drugs, hirudin and bivalirudin, allowed for only a partial control of the PCA of hALPCs (256.3±11.8 sec [n = 3] and 377.7±107.2 sec [n = 6], respectively, p<0.01 and p<0.001 as compared to control respectively) (Figure 4B), even when increasing the dose by two or five times (Figure S3 and S4). Hepatocyte PCA was controlled by unfractionated heparin, low molecular weight heparin, the pentasaccharide fondaparinux (Figure 4C) and the direct thrombin inhibitor drugs, hirudin, and bivalirudin (Figure 4D). A control blood sample in the absence of cells had a CT of 1075.0±107.2 (n = 6) when bivalirudin was added, while no measurable coagulation was observed with hirudin.

Anti-vitamin K drugs (using plasma from patients on long-term anticoagulation when the International Normalized Ratio (INR) was steady between 2 and 3) had no influence on the thromboelastometry, even for controls with the absence of cells (data not shown).

**Figure 5:**
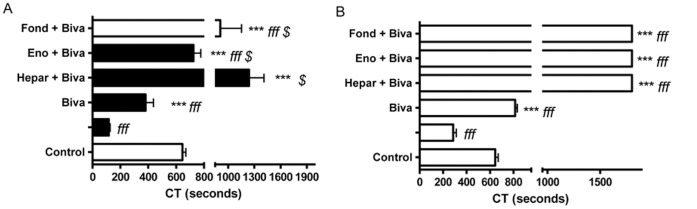
Modulation of hALPCs PCA by combination of anticoagulants. Figure 5A Clotting time (CT) assayed by ROTEM after recalcification, with added tissue factor (ExTem 20 µL) of citrated whole blood (300 µl) in presence or not of human adult liver progenitor cells (hALPCs) suspended in human albumin 5% with heparin (Hepar), enoxaparin (Eno), or fondaparinux (Fond) extemporaneously added to blood. Combination of anticoagulant drugs was obtained when bivalirudin (Biva) was extemporaneously added to blood. hALPCs (black), Control (albumin) (grey). * as compared to hALPCs *f* as compared to control $ as compared to bivalirudin Figure 5B Clotting time (CT) assayed by ROTEM after recalcification, with added tissue factor (ExTem 20 µL) of citrated whole blood (300 µl) in presence or not of hepatocytes suspended in human albumin 5% with heparin (Hepar), enoxaparin (Eno), or fondaparinux (Fond) extemporaneously added to blood. Combination of anticoagulant drugs was obtained when bivalirudin (Biva) was extemporaneously added to blood. Hepatocytes (white), Control (albumin) (grey). * as compared to hepatocytes *f* as compared to control $ as compared to bivalirudin.

Finally, the concomitant use of bivalirudin with unfractionated heparin (1240.0±338.7 sec [n = 3], p<0.05 as compared to bivalirudin alone), enoxaparin (725.0±90.1 sec [n = 3], p<0.05 as compared to bivalirudin alone), or fondaparinux (909.0±421.4 sec [n = 3], p<0.05 as compared to bivalirudin alone) was shown to be a synergic combination with antithrombin activator and thrombin inhibitor, allowing the PCA of hALPCs to be modulated (Figures 5A and B). However, no complete modulation of the PCA of hALPCs was obtained when combining heparin with enoxaparin or fondaparinux (Figure S5).

We also demonstrated the potential of combining of bivalirudin with a direct anti-thrombotic agent targeting factor-Xa (Rivaroxaban), while the use of rivaroxaban alone was ineffective on hALPCs PCA (Figure S6).

Using analogous experiments, we demonstrated that unfractionated heparin was able to control the PCA of bone marrow mesenchymal cells and skin fibroblasts, but that it remained inactive on liver myofibroblast PCA (Figure S7). In addition, the concomitant use of unfractionated heparin and bivalirudin was shown to modulate the PCA of liver myofibroblasts in contrast with bivalirudin alone (Figure S8).

### Comprehension of the PCA of hALPCs

#### hALPCs express TF and TFPI

**Figure 6:**
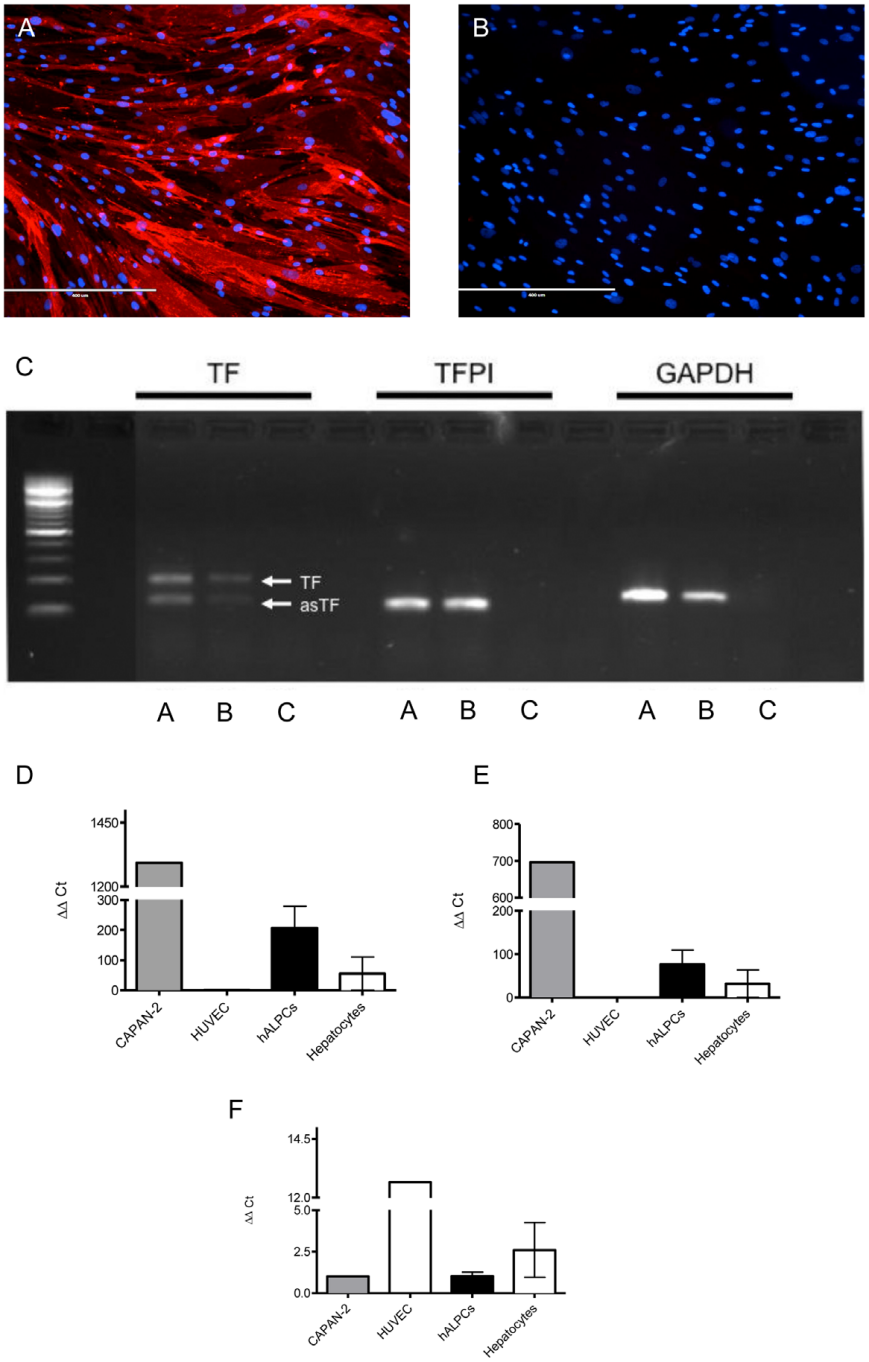
TF expression. Figure 6A–B. Tissue factor (TF) expression in hALPCs. Immunofluorescence for TF was performed on human adult liver progenitor cells (hALPCs) placed on coverslips and fixed by paraformaldehyde (magnification 100×) (A). The nuclei were revealed by DAPI (blue staining). As a negative control, immunofluorescence was performed on hALPCs without primary antibody (B). Figure 6C Tissue factor (TF) and tissue factor pathway inhibitor (TFPI) mRNA expression in human adult liver progenitor cells (hALPCs) and hepatocytes evaluated using conventional reverse transcription polymerase chain reaction. TF, alternatively spliced TF (as-TF), TFPI, and glyceraldehyde 3-phosphate dehydrogenase (GAPDH) (technical control). A) hALPCs, B) Hepatocytes, C) Control Figure 6D–E–F Tissue factor mRNA (TF and as-TF), and tissue factor pathway inhibitor (TFPI) mRNA expression of human adult liver progenitor cells (hALPCs) and hepatocytes evaluated using real-time polymerase chain reaction. Semi-quantitative expression of the mRNA of the TF gene (A), as-TF (B), and the TFPI gene (C) among hALPCs cells and hepatocytes. CAPAN-2 cells and HUVEC are the positive control for TF, as-TF, and TFPI.

TF expression was first documented using immunofluorescence. As shown in Figure 6A–B, we found that all cells expressed TF constitutively (uniform cytoplasmic staining). Flow cytometry analysis of hALPCs confirmed a positive specific staining for TF (94.9±1.0% for membrane bound form and 93.6±10.2% for cytosolic form as compared to control isotype 24.2±6.1% and 7.6±5.8%, respectively, and unmarked cells 13.2±7.1% and 3.7±4.1%, respectively; n = 3).

The expression of TF and tissue factor pathway inhibitor (TFPI) was assessed at the mRNA level using reverse transcription polymerase chain reaction (RT-PCR) (Figure 6C). Both the membrane form and alternatively spliced variant of TF mRNA were expressed in hALPCs, as was TFPI. In further experiments, we used real-time RT-PCR to quantify TF, alternatively spliced TF (as-TF), and TFPI mRNA levels. As shown in Figure 6D–E–F, the membrane TF was predominantly expressed (n = 3). Furthermore, the expression of TF was higher in hALPCs compared to hepatocytes (n = 3), whereas the expression of TFPI in hepatocytes was higher than in hALPCs (n = 3).

The role of TF in the induction of PCA was determined by the pre-incubation of cells with anti-human TF IgG at a concentration of 0.2 mg/ml. The PCA of hALPCs was partially controlled by blocking TF (324.8±11.4 sec [n = 5], p<0.01 compared to without the TF antibody), which was in contrast to hepatocytes, as previously demonstrated (Figure S9) (7).

As shown in Figure 4, only a partial control of the PCA of hALPCs was obtained in factor VII deficient plasma, possibly related to the fact that small amounts of residual factor VII were sufficient to induce coagulation in presence of hALPCs.

#### HALPCs and heparin

Only minor anti-Xa activity was observed in plasma obtained after the incubation of hALPCs (0.05±0.03 UI/ml) and heparin at a concentration of 10 UI/ml (Figure S10), which correlated with the absence of anticoagulant effect of heparin alone in hALPCs.

### Clinical Applications and Anticoagulation Protocol

**Figure 7:**
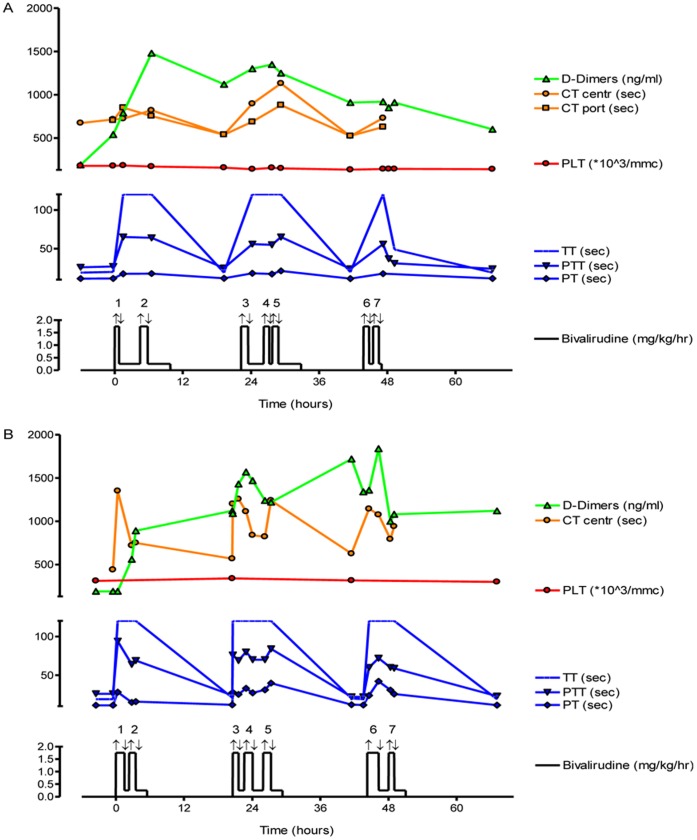
HALPCs infusion and anticoagulation protocol. During cell infusion, patients received bivalirudin (1.75 mg/kg). Between consecutive cell infusions, the dose was decreased to 0.25 mg/kg for 2 to 4 hours. Coagulation tests were repetitively performed before each infusion, 20 min after beginning and at the end, and included the following: thromboelastometry with clotting time (CT) in the portal vein (port) or via the central line (centr); platelets (PLT) (normal values: 150–350 10exp3/µl); D-dimer levels (normal values: <500 ng/ml), thrombin time (TT) (normal values: 15–24 sec); prothrombin time (PT) (normal values: 9–14 sec); partial thromboplastin time (PTT) (normal values: 20–33 sec). A Crigler Najjar patient. B Glycogenosis Type 1a patient.

The anticoagulation protocol was successfully applied, as no thrombotic or haemorrhagic events occurred in the two patients. With bivalirudin use, a substantial increase was observed in thrombin time (TT), whereas the prothrombin time (PT) remained virtually unchanged. A small increase in D-dimer levels was noted in both patients, reaching 1480 ng/ml for the first patient and 1840 ng/ml for the second. An increase in partial thromboplastin time (PTT) was detected in both patients, which correlated with detectable anti-Xa activity (Figure 7A and B). There was no modification in portal flow using liver Doppler ultrasound during the infusions of both patients.

## Discussion

Vein thrombosis at the site of infusion is a potential and major complication in human cell-based therapies. Our present study shows the thrombogenic risk of human mesenchymal cells, as related to the PCA of these cells. We first demonstrated that both hALPCs and bone marrow mesenchymal stem cells exhibited significantly measurable PCA. This was further confirmed for other cells of mesenchymal phenotype like skin fibroblasts and liver myofibroblasts (activated stellate cells). The risk of thrombosis related to the infusion of hALPCs was then shown not to be controlled with antithrombin activator (Heparins, Fondaparinux), direct factor Xa inhibitor (Rivaroxaban) or thrombin inhibitor (Hirudin, Bivalirudin) alone. Instead, an innovative specific anticoagulant protocol acted synergistically to counter this PCA. This dual antithrombotic therapy effectively prevented the risk of thrombosis in human clinical practice.

By revealing the PCA of mesenchymal cells, our study confirmed the previous data from animal studies in which intra-arterial mesenchymal stem cell infusion led to the occlusion of distal vasculature. This result was attributed to the relatively large cell size, and the authors consequently recommended that mesenchymal stem cells be used cautiously when infused via the intravascular route [15]. We demonstrated the procoagulant potential of the cell itself independently of its size.

PCA and subsequent thrombosis events would cause, besides bloodstream modifications, a cell loss and reduction in cell engraftment, thus impairing the final efficacy of cell transplantation [10]–[13]. We thus investigated in-depth the PCA of hALPCs, as these cells are candidates for curing metabolic diseases in humans [14], [16], [17]. PCA was determined using thromboelastometry with or without extrinsic TF addition, which is a viscoelastometric method for haemostasis testing in whole blood, measuring the interaction of coagulation factors, inhibitors, and cellular components during the clotting phase and subsequent clot lysis. The rheological conditions of this method mimic the sluggish flow of blood in the veins. The different parameters in thromboelastometry reflect the synergistic activity of the plasma coagulation system, platelet function, and fibrinolysis. A limitation of thromboelastometry is that it only measures part of the process of thrombin generation. It does not take into account the eventual inhibition of thrombin by the natural anticoagulants.

In our study, the PCA of hALPCs was revealed in blood as well as plasma, suggesting that platelet activation did not play a role in this mechanism. The PCA of hALPCs was further confirmed by the tubing loop method. The more pronounced PCA of hALPCs compared to hepatocytes may be explained by the increased TF expression and decreased TFPI expression. As reported in the literature, fibroblasts, vascular smooth muscle cells, and cardiac myocytes, which are all mesenchymal cells, express small amounts of cell-surface TF under physiologic conditions. Indeed, under normal conditions, TF expression is confined to the extravascular sites, separated from the circulating blood by a tissue barrier, which, when disrupted, allows plasma factor VII/VIIA to be exposed to TF and initiate clotting. In this study, TF expression of these extravascular space cells, TF mRNA and antigen, were increased between eight- and ten-fold by serum stimulation in culture [18]. TF expression is also increased by other growth factors, PDGF and EGF, or by non mitogenic agents (bacterial LPS) [19]. In our study, hALPCs were cultured in serum, with cells at passages 4 to 6 being examined, as these may increase TF expression.

We also incubated cells with anti-TF antibody at a concentration inhibiting HUVEC and hepatocytes PCA to ascertain the role of TF in inducing PCA of hALPCs. We only obtained a partial control of hALPCs PCA while using this antibody. This may be related to a non-saturating concentration of the antibody to counteract the increased TF expression by hALPCs. We also only obtained a partial control of hALPCs PCA by using factor VII deficient plasma. To interpret these results, we have to consider that factor VII deficient plasma may contain a small amount of factor VII, and that coagulation may have been induced when TF, derived from cells, was exposed to even a small amount of factor VII.

Surprisingly, the PCA of hALPCs and liver myofibroblasts was not inhibited by unfractionated heparin alone, which is not the case for bone marrow mesenchymal stem cells and skin fibroblasts. The anticoagulant effect of unfractionated heparin predominantly acts by binding to and increasing the natural anticoagulant activity of antithrombin and TFPI [20]. Indeed, the release of TFPI into plasma is induced by heparins but is the lowest for unfractionated heparin as compared to low molecular weight heparin [20]. Unfractionated heparin is considered the most important anticoagulant drug used in hepatocyte transplantation, being added to the infusion medium. The absence or low level of anti-Xa activity as measured in plasma in contact with hALPCs and unfractionated heparin (except for high doses) may suggest a particular interaction between cells and heparin.

Heparin is also known to inhibit the proliferation and TF expression of smooth muscle cells in various vascular smooth muscle cell tissue cultures [19], [21], [22]. The exact mechanism by which heparin inhibits TF expression is still to be elucidated, but it may be triggered after binding of heparin to a receptor [23]–[25]. To explain the absence of an effect of heparin on the PCA of hALPCs, we may hypothesise that heparin is attached to the binding domain on the cell surface. Heparin is linked to the cell, being the starting point of several reactions aimed at decreasing the proliferation of the cell and TF expression, and thus limiting its availability for a significant blockade of the hALPCs PCA. In addition, hALPCs can also neutralise heparin, but not by specifically using ionic charges. Furthermore, data from Xuereb et al. demonstrated that two downstream pathways could be activated by TF in smooth muscle cells: one PKC dependent, heparin insensitive elicited by LPS, and the other ERK dependent, heparin sensitive elicited by the mitogenic agents but uncoupled to the pathway for proliferative response [19]. Additionally, other antithrombin activators, such as low molecular weight heparin (enoxaparin) or fondaparinux, or direct factor Xa inhibitor did not inhibit the PCA of hALPCs when used alone and even at high concentrations.

Following our observation that PCA was absent when using factor II deficient plasma, we tested the capacity of the direct thrombin inhibitors, hirudin and bivalirudin, in inhibiting PCA in our model. It is also known that direct thrombin inhibitors inhibit clot-bound thrombin more potently than heparins [26]. The PCA of hALPCs was only partially inhibited by these direct thrombin inhibitors. In this context, a recent paper demonstrated that stimulation of human smooth muscle cells with thrombin as well as factor VIIa/factor X led to a significant induction of both TF isoforms on mRNA and protein levels, confirming data from the literature [27], but also to increased TF activity in a chromogenic assay [28]. In contrast to the thrombin-stimulated TF isoforms expression and TF activity, treatment with bivalirudin had no impact on factor VIIa/factor X-induced upregulation of TF and on increased TF activity in smooth muscle cells. These important results showed that increased TF expression and activity differed if obtained after thrombin or factor VIIa/factor X stimulation as bivalirudin has no effect on factor VIIa/factor X TF expression and activity [28]. This is in line with our results that showed that the combination of antithrombotic agents targeting IIa and Xa is needed to control hALPCs PCA, related to increased TF expression. As discussed earlier, two different pathways are probably responsible for increased TF expression, one heparin sensitive and the other heparin insensitive and thus maybe the bivalirudin sensitive one.

Combination of a low molecular weight heparin and recombinant hirudin was found to reduce successfully the thrombus growth in a rabbit jugular vein thrombosis model [29]. Finally, it was also shown experimentally that following hirudin treatment discontinuation, a hypercoagulation rebound occurred, possibly related to the persistence of factor Xa activity in the clot. The authors subsequently demonstrated the complementary effect of DX9065a, a clot-bound factor Xa inhibitor, and r-hirudin, which increased the antithrombotic effect in an in-vitro model for the measurement of clot-bound thrombin [30]. The combination of an antithrombin activator and thrombin inhibitor was used in a recent study on patients with ST-elevation myocardial infarction, resulting in a reduction in mortality or definite thrombosis following primary percutaneous angioplasty [31]. Bivalirudin has a short half-life of 35 to 40 minutes and has a reversible effect, while hirudin has a non-reversible effect and requires novel thrombin synthesis in order to return to normal haemostasis status. Bivalirudin use in children was previously evaluated using an adult dosage (0.75 mg/kg bolus and 1.75 mg/kg/hr infusion), and safely provided the expected anticoagulant effect in a paediatric population undergoing intravascular procedures for congenital heart disease [32]. For clinical application, we chose unfractionated heparin, typically used in hepatocyte transplantation and easily added to the infusion medium, and bivalirudin given its short half-life and available paediatric data.

We successfully tested this anticoagulation protocol in two hALPC-transplanted recipients, without any side effects being observed during or following cell transplantation.

The high increase in D-dimer levels that was observed in previous patients who experienced partial thrombosis was not found in our two patients, confirming the antithrombotic efficacy of this anticoagulation protocol. Furthermore, PCA not only causes local thrombosis and microthrombosis, but also induces local inflammation, which may lead to cell rejection. Thus, the proposed anticoagulation combination may optimise engraftment and repopulation by hALPCs.

To conclude, the original combination of two anticoagulant drugs, an antithrombin activator or a direct factor Xa inhibitor and a thrombin inhibitor, can reduce or even prevent the risk of thrombosis, which is associated with intravascular infusion of cells in humans. This anticoagulation strategy has probably also an interest for islet transplantation where TF expression by islets and duct cells plays also a pivotal role in inducing PCA and related instant blood mediated inflammatory reaction. By preventing thrombosis, microthrombosis, and inflammation, this anticoagulation should also optimise the safety and success of cell, pancreatic islet and stem cell transplantation in humans.

## Methods

### Ethics Statement

The protocol, including all experiments on human samples, as well as the human off-label anticoagulant protocol use and informed consent were approved by the institutional ethics review board (Comission d’éthique biomédicale hospitalo-facultaire, Université Catholique de Louvain, Faculté de Médecine, commission.ethique@md.ucl.ac.be, chaired by J.M. Maloteaux). Written informed consent was obtained from the patient (If applicable) and from the next of kin.

### Cell Preparations

hALPCs were obtained from healthy liver donors (n = 6, aged 9 to 44 years) as previously described [14). We studied freshly trypsinised cells or cells after cryopreservation/thawing at passages 4 to 6, with a viability exceeding 90% at the trypan blue test. Cells were suspended in an albumin solution with or without heparin at a concentration of 10 U/mL (or more when specified). As a control, cryopreserved/thawed human hepatocytes (n = 5, aged 16 to 44 years) were used. Liver isolation and hepatocyte cryopreservation/thawing procedures were previously published in detail [4].

Bone marrow samples were collected by the aspiration of vertebrae or iliac crests of three post-mortem organ donors aged 8 to 67 years. Aspirates were collected into heparinised syringes containing 10% Hanks’ balanced salt solution (Invitrogen, Merelbeke, Belgium) and processed within 48 hours according to a previously described protocol [33].

Human fibroblasts were collected from a skin biopsy (medio-anterior side of the forearm) of three volunteers aged 18 to 35 years after obtaining written informed consent as previously described [34].

Human liver non-parenchymal cells were obtained after liver isolation was performed in our tissue bank, involving filtration and two low-speed centrifugations of the cell suspension from three different donors (one neonate liver and two 12-years-old donors). Next, human stellate cells were isolated using Nycodenz gradient centrifugation (Myegaard, Oslo, Norway) according to established protocols and in collaboration with the Department of Cell Biology-VUB (Prof. LA van Grunsven) [35]. Activated myofibroblasts were obtained from the isolated stellate cells.

### Blood

Blood was obtained from five male donors aged 29 to 40 years.

### Procoagulant Activity of hALPC Suspension

Measurements were performed on a ROTEM® delta analyser (Pentapharm, Munich, Germany). ROTEM® assessed the kinetics and quality of clot formation and clot lysis in real time. Clotting time (CT) was defined as the period of time from the start of analysis until the start of clot formation, normally until 2-mm amplitude was reached. Clot formation time was classified as the period until 20-mm amplitude was reached. The alpha angle was defined as the angle between the centre line and a tangent to the curve through the 2-mm amplitude point, which was at the end of CT. The maximum amplitude of the curve represented the maximum clot firmness, while the maximum of lysis the maximum fibrinolysis detected during the measurement. Our analysis focused on CT.

In short, after a brief rest period, 300 µl of whole blood was pipetted into a cup preheated to 37°C. Suspended cells were subsequently added to the whole blood (five 10exp5 cells if not specified), with 20 µl of trigger reagent containing tissue factor (Innovin, Siemens, Marburg, Germany; final dilution 1∶17000/0.35 pM) diluted in Owren buffer (Siemens, Marburg, Germany) then being added to the cell-blood mixture followed by the necessary addition of 20 µl of 0.2 M CaCl_2_. After adding calcium, measurements were initiated automatically. The PCA of cells was also determined without adding Innovin. Thus, in order to ascertain the role of TF in this coagulation model, cells were pre-incubated at room temperature for 10 min with either 0.2 mg/mL anti-human TF IgG1 monoclonal antibody (mAb) (American Diagnostica) or 0.2 mg/mL mouse IgG1 mAb (clone11711.11; RnD Systems, Abingdon, United Kingdom) before extensive washing with albumin 5% followed by the thromboelastometry assay, concentration inhibiting HUVEC and hepatocytes PCA [8], [36].

For plasma assays, cells (five 10exp5 cells if not specified) were incubated in 3.8 ml of citrated blood at 37°C for 30 minutes. After incubation, whole blood was centrifuged at 4500 rpm for 10 minutes. Consequently, 300 µl of the obtained plasma was ready for the protocol, pipetted into the cup with the addition or not of Innovin and CaCl_2_.

For factor-deficient plasma assays, suspended cells (five 10exp5 cells if not specified) were combined with 300 µl of plasma before adding Innovin and CaCl_2_.

For the modulation of PCA assays, cells were suspended in albumin 5% with or without unfractionated heparin (Heparin Leo®, Leo) at a concentration of 10 UI/ml. The following were then added to blood or plasma: low-molecular-weight heparin, enoxaparin (Clexane®, Aventis Pharma) at a concentration of 1 UI/ml following published data [37], pentasaccharide antithrombin activator (Fondaparinux, Arixtra®, GSK) at a concentration of 0.34 mg/l and dose extrapolation of 2.5 mg/kg for adults following published data [38], rivaroxaban (Xarelto®, Bayer Schering) at a concentration of 1 µg/ml following published data [39], hirudin (Refludan®, Celgène Europe Limited) at a concentration of 5.7 µg/ml and dose extrapolation of 0.4 mg/kg, and bivalirudin (Angiox®, The Medicines Company) at a concentration of 10.7 µg/ml and dose extrapolation 0.75 mg/kg. Dose extrapolation was based on circulating blood volume according to weight (70 ml/kg).

If no coagulation was observed after 1800 sec, thromboelastometry was arbitrarily stopped.

### Tubing Loop

A whole-blood experiment protocol was adapted from a model previously described [12]. Loops made of polyvinyl chloride tubing (inner diameter 6.3 mm, length 390 mm) and treated with a Corline heparin surface were purchased from Corline (Uppsala, Sweden). Loops were supplemented with cell samples (five 10exp5) suspended in phosphate buffered saline before adding blood. Thereafter, 5 mL of non-anticoagulated blood from healthy volunteers was added to each loop. To generate a blood flow of approximately 45 mL/minute, loop devices were placed on a platform rocker inside a 37°C incubator. Blood samples were collected in tubes containing ethylene diamine tetraacetic acid (4.1 mmol/L final concentration) and citrate (12.9 mmol/L final concentration) before and 30 minutes after the start. Platelets were counted by the XE-2100 automate haematology analyser (Sysmex, Japan), and D-dimer levels were evaluated using the immunoturbidimetric assay (Innovance D-Dimer, Siemens, Marburg, Germany) on a CA-7000 system (Sysmex, Japan).

### Anti-Xa Activity Measurement

Anti-Xa activity measurement was performed using the Biophen Heparin (LRT) kit adapted on a CA7000 (Siemens, Marburg, Germany). In short, the assay was a chromogenic kinetic method based on the inhibition of a constant amount of factor Xa by the tested heparin (or other anti-Xa substance) in the presence of endogenous antithrombin as well as on the hydrolysis of a factor Xa specific chromogenic substrate by the factor Xa in excess. After a 30-min incubation of the cells suspended in albumin with or without heparin (10 UI/ml, 50 UI/ml, and 100 UI/ml) in the blood, anti-Xa activity was measured in plasma obtained following blood centrifugation.

### TF and TFPI Expression of hALPC Suspension

Immunofluorescence studies were performed in order to evaluate the presence of TF. Thus, human adult liver-derived stem cells were placed on coverslips and fixed with paraformaldehyde 4% (Merck, Darmstadt, Germany) for 20 minutes. These cells were then incubated with Triton X-100 (Sigma,Bornem, Belgium) 1% in Tris-base sodium buffer (50 mmol/L Tris-HCl pH 7.4 and 150 mmol/L NaCl) (Organics [VWR], Leuven, Belgium) for 15 minutes and then with 3% milk in Tris-base sodium buffer for 1 hour. The primary antibody, murine mAb anti-TF IgG1 (immunoglobulin [Ig]G1 n4508; American Diagnostica, Andresy, France), was diluted (1/50) in Tris-base sodium and incubated with the cells for 1 hour. The secondary antibody was fluorescein isothiocyanate-conjugated anti-mouse IgG (Sigma). The nuclei were revealed with 4_, 6-diamidino- 2-phenylindole staining (DAPI; Sigma). Negative experimental controls were performed relating to the absence of primary or secondary antibodies. The presence of TF was also confirmed by flow cytometric analysis. In order to detect the membrane-bound form of TF, cells were washed in phosphate buffered saline supplemented with 0.5% bovine serum albumin (FACS buffer) and incubated for 20 minutes at 4°C with the fluorescein isothiocyanate-conjugated IgG1 mAb against TF no. 4508CJ (American Diagnostica) or the corresponding isotype-matched control mAb (BD Biosciences, Erembogedem, Belgium) diluted in FACS buffer containing 10% decomplemented pooled human serum. In order to detect the cytosolic form of TF, cells were incubated with Cytofix/Cytoperm (BD Biosciences) for 20 min at room temperature and washed with Perm/Wash (BD Biosciences).The samples were then incubated for 20 min at room temperature with fluorescein isothiocyanate-conjugated anti-TF mAb or the corresponding isotype-matched control mAb (BD Biosciences) diluted in Perm/Wash. Cell fluorescence was measured using a BD FACS CANTO II flow cytometer and analysed with the BD FACS Diva software.

No anti-TFPI antibody was obtained to evaluate the TFPI expression using immunocytochemistry or flow cytometry analysis.

The two forms of TF and TFPI were analysed by reverse-transcription polymerase chain reaction (RT-PCR). Messenger ribonucleic acid (mRNA) was extracted from 0.5×10^6^ cells using the TriPure isolation reagent kit (Roche Applied Science, Brussels, Belgium) according to the manufacturer’s instructions. One-step RT-PCR was performed on a Thermocycler instrument (Applied Biosystems, Lennik, Belgium) with primers synthesised at Invitrogen. RT-PCR for TF or glyceraldehyde 3-phosphate dehydrogenase was conducted with the primers detailed in Table 1.

**Table 1:** TF, TFPI and GAPDH primers.

Primer	Sequence
TF sense primer	5-TGAATGTGACCGTAGAAGATGA-3
TF antisense primer	5-GGAGTTCTCCTTCCAGCTCT-3
as-TF sense primer	5-TCTTCAAGTTCAGGAAAGAAATATTCT-3
as-TF antisense primer	5-CCAGGATGATGACAAGGATGA-3
TFPI sense primer	5-GGAAGAAGATCCTGGAATATCGAGG-3
TFPI antisense primer	5-CTTGGTTGATTGCGGAGTCAGGGAG-3
GAPDH sense primer	5-CGGACTCAACGGATTTGGTCGTAT-3
GAPDH antisense primer	5-AGCCTTCTCCATGGTGGT-3

TF: tissue factor; as-TF: alternatively spliced tissue factor; TFPI: tissue factor pathway inhibitor; GAPD: glyceraldehyde 3-phosphate dehydrogenase.

Products were separated by electrophoresis on 1% agarose gel and visualised with ethidium bromide staining and ultraviolet illumination.

Real-time RT-PCR for TF, as-TF, TFPI, and cyclophilin A was carried out using the StepOnePlus Real-Time PCR system (Applied Biosystems, California, USA) with TaqMan® Gene Expression Assays as listed in Table 2. For TF expression, two assays were used, with one (common TF) amplifying a region present in both membrane and soluble forms (as-TF), and the other (membrane TF) amplifying a region present only in membrane form (standard). The Ct (threshold cycle) parameter was derived for each cDNA sample and primer pair, with Cyclophilin A Ct being subtracted in order to obtain the ΔCt. ΔΔCt was then obtained by subtracting the Ct calibrator gene, with the results expressed as the fold change of the mRNA amount (Figure 6D–E–F). The as-TF expression was calculated as the difference between the ΔΔCt of the common TF and membrane TF. The primers are detailed in Table 2.

**Table 2:** References for real-time polymerase chain reaction.

Gene	TaqMan® GeneExpression Assays	Amplicon length
as-TF	Hs01076032_m1	69
TF membrane	Hs01076029_m1	85
TFPI	Hs01041344_m1	78
Cyclophilin A	Hs99999904_m1	98

TF: tissue factor; as-TF: alternatively spliced tissue factor; TFPI: tissue factor pathway inhibitor.

CAPAN-2 cell line was used as a TF positive control, while HUVEC cell line as a TFPI positive control.

**Table 3:** Coagulation tests.

Coagulation test	
D-dimers level	D-dimer is a fibrin degradation product, a small protein fragment present in the blood after a blood clot is degraded by fibrinolysis. It is so named because it contains two crosslinked D fragments of the fibrinogen protein
Thrombin Time (TT)	The Thrombin Time is a blood test which measures the time it takes for a clot to form in the plasma of a blood sample anticoagulant to which an excess of thrombin has been added. This test is repeated with pooled plasma from normal patients. The difference in time between the test and the ‘normal’ indicates an abnormality in the conversion of fibrinogen (a soluble protein) to fibrin an insoluble protein.
Prothrombin time (PT) and International Normalized Ratio (INR)	The Prothrombin time and its derived measures of prothrombin ratio and international normalized ratio (INR) are measures of the *extrinsic pathway* of coagulation. They are used to determine the clotting tendency of blood, in the measure of warfarin dosage, liver damage, and vitamin K status. PT measures factors I, II, V, VII, and X.
Partial Thromboplastin Time (PTT)	The Partial Thromboplastin time or activated Partial Thromboplastin Time is a performance indicator measuring the efficacy of both the “intrinsic” (now referred to as the contact activation pathway) and the common coagulation pathways. Apart from detecting abnormalities in blood clotting, it is also used to monitor the treatment effects with heparin, a major anticoagulant. Kaolin Cephalin Clotting Time is a historic name for the activated Partial Thromboplastin Time.

### Infusions of Patients and Anti-coagulation Protocol

A 3-year-old girl, suffering from severe ornithine transcarbamylase deficiency (<1% activity), was the first recipient of hALPCs. The diagnosis was established 12 days after birth and confirmed by DNA analysis, which indicated a *de novo* mutation of exons 6 and 8 on the paternal allele of ornithine transcarbamylase gene.

The girl received from a male donor two separate infusions of 30 million hALPCs per kg of body weight, with a 2-week interval between infusions. In total, the patient received 0.9 billion progenitor cells. The cells were suspended in albumin 5% and unfractionated heparin (10 UI/ml).

The first hALPC infusion was performed under general anaesthesia without any premedication. A transcutaneous catheter was placed in the main portal vein branch under fluoroscopy and ultrasound guidance after injecting one dose of Cefazolin (40 mg/kg). Cell infusion was performed using a syringe of 50 ml at a flow rate of 100 cc/h. Immune suppression was administrated to the patient using tacrolimus (Prograft®, Astellas Pharma) with the monotherapy (0.1 mg/kg) corresponding to 2 mg per day in two divided doses to reach levels of 6–7 ng/ml. Cefazolin (40 mg/kg) was administrated as prophylactic antibiotherapy twice post-infusion with an 8-hour interval between the two doses. The infusion and post-infusion periods were unremarkable, with the child being discharged from hospital on Day 3 post-infusion.

The second hALPC infusion was performed two weeks later. In the interim, a partial thrombosis of the intra-hepatic portal vein branch had occurred and led to stopping the infusion. This adverse event was treated with heparin and coumarinic anticoagulant (5 mg/day).

D-dimer levels were markedly elevated after both courses of cell infusion. This adverse event was without consequence for the patient, but justified a further investigation of the procoagulant effect of progenitor cells.

The second patient was a 24-year-old man with intermediate type I/II Crigler-Najjar syndrome, which did not respond to phenobarbital. The diagnosis was established 1 month after birth and confirmed by DNA analysis, indicating a mutation on the UDP-glucuronosyltransferase 1A1 gene with the presence of the homozygous state for the L443P mutation. The patient received 2.2 billion hALPCs administered in seven infusions over 2 days. Prior to the placement of the portal catheter, the patient was administered premedication, including cefazolin (1 gr). The catheter was under ultrasound control in the portal system. Solumedrol (80 mg) was injected before the infusion. The immunosuppression treatment consisted of tacrolimus (Prograft®, Astellas Pharma), which targeted blood levels of 6–8 ng/ml. A specific coagulation prophylaxis was prescribed, with cells being suspended in albumin 5% and heparin at a concentration of 10 UI/ml. During cell infusion, the subject received bivalirudin (1.75 mg/kg) intraveinously by continuous infusion, while between consecutive cell infusions, he was given bivalirudin (0.25 mg/kg) for 2 to 4 hours depending on the thromboelastometry test. Coagulation tests including thromboelastometry (CT), platelet counts (normal values: 150–350 10exp3/µl), D-dimer levels (normal values: <500 ng/ml), thrombin time (TT, normal values: 15–24 sec), prothrombin time (PT, normal values: 9–14 sec), and partial thromboplastin time (PTT, normal values: 20–33 sec) were repetitively performed before each infusion, 20 min after the beginning, and at the end. A liver Doppler ultra-sound was conducted after each infusion to assess portal flow. The signification of coagulation tests was summarised in table 3.

The third patient was a 17-years-old suffering from glycogenosis type 1a, as documented by genetic analysis (G188R mutation and 380insC insertion) and the absence of glucose-6 phosphatase activity on a liver biopsy. The patient also received antibiotic prophylaxis prior to the placement of the portal catheter as well as steroids before the infusion. The same immunosuppressor regimen as patient 2 was administered. The patient received 3 billion progenitor cells administered in seven infusions over 3 days aimed at controlling recurrent hypoglycemia. The same anticoagulation protocol and coagulation, including liver Doppler ultrasound follow-up, was applied.

### Statistics

Mann-Whitney tests were used to assess statistically significant differences (*P<0.05, **P<0.01, ***P<0.001). Any significant values were adjusted according to the Bonferroni correction in order to avoid Type 1 errors. The Kruskal-Wallis test was used for one-way ANOVA analysis.

## Supporting Information

Figure S1 **Supernatant of hALPCs PCA.** Clotting time (CT) essayed by ROTEM after recalcification, with added Tissue Factor (ExTem 20 µL), of citrated whole blood (300 µl) in presence of supernatant of hALPCs culture. No coagulation is induced if absence of recalcification.
(docm)

Figure S2 **Modulation of hALPCs PCA by heparin.** A) Clotting time (CT) assayed by ROTEM after recalcification, with added tissue factor (ExTem 20 µL) of citrated whole blood (300 µl) in presence or not of human adult liver progenitor cells (hALPCs) (Black) suspended in human albumin 5% and with or without heparin (Hepar) at several concentrations (Hepar-10 UI/ml, Hepar 5×−50 UI/ml, and Hepar 10×−100 UI/ml) or not Control (albumin) (grey) *f* as compared to control. B) Clotting time (CT) assayed by ROTEM after recalcification, with added tissue factor (ExTem 20 µL) of citrated whole blood (300 µl) in presence or not of human adult liver progenitor cells (hALPCs) (Black) suspended in human albumin 5% and with or without fondaparinux (Fond) and enoxaparin (Eno) at normal concentrations or increased five times (5×) the normal concentration Control (albumin) (grey) *f* as compared to control Fond *vs.* Fond 5×, non-significant Eno *vs.* Eno 5×, non-significant.
(docm)

Figure S3 **Modulation of hALPCs PCA by hirudin.** Clotting time (CT) assayed by ROTEM after recalcification, with added tissue factor (ExTem 20 µL) of citrated whole blood (300 µl) in presence or not of human adult liver progenitor cells (hALPCs) suspended in human albumin 5%. Increased concentrations of hirudin (Hir) at two (Hir 2x) or five times the normal levels (Hir 5x)) was extemporaneously added to blood. hALPCs (black), Control (albumin) (grey). * as compared to hALPCs *f* as compared to control hALPCs Hir *vs.* hALPCs Hir 2x, n.s.
(docm).

Figure S4 **Modulation of hALPCs PCA by bivalirudin.** Clotting time (CT) assayed by ROTEM after recalcification, with added tissue factor (ExTem 20 µL) of citrated whole blood (300 µl) in presence or not of human adult liver progenitor cells (hALPCs) suspended in human albumin 5%. Increased concentrations of bivalirudin (Biva) two times the normal level (Biva 2x) was extemporaneously added to blood. hALPCs (black), Control (albumin) (grey). * as compared to hALPCs *f* as compared to control.
(docm)

Figure S5 **Modulation of hALPCs by antithrombin activators in combination.** Clotting time (CT) assayed by ROTEM after recalcification, with added tissue factor (ExTem 20 µL) of citrated whole blood (300 µl) in presence or not of human adult liver progenitor cells (hALPCs) suspended in human albumin 5% and with or without heparin (Hepar). Enoxaparin (Eno) or fondaparinux (Fond) was extemporaneously added to blood with cells suspended in heparin or not hALPCs (black), Control (albumin) (grey) *f* as compared to control.

Figure S6 **Modulation of hALPCs by direct inhibition of factor X in combination or not with bivalirudin.** Clotting time (CT) assayed by ROTEM after recalcification, with added tissue factor (ExTem 20 µL) of citrated whole blood (300 µl) in presence or not of human adult liver progenitor cells (hALPCs) suspended in human albumin 5% with rivaroxaban. Combination of anticoagulant drugs was obtained when bivalirudin (Biva) was extemporaneously added to blood. hALPCs (black), Control (albumin) (grey). * as compared to hALPCs *f* as compared to control $ as compared to bivalirudin.
(docm)

Figure S7 **Modulation of mesenchymal cells PCA by heparin.** Clotting time (CT) assayed by ROTEM after recalcification, with added tissue factor (ExTem 20 µL) of citrated whole blood (300 µl) with human adult liver progenitor cells (hALPCs), hepatocytes, skin fibroblasts, bone marrow mesenchymal stem cells (BMMSC), bone marrow haematopoietic stem cells (BMHSC), or liver myofibroblasts suspended in human albumin 5% with or without heparin (10 UI/ml) (Hepar) *f* as compared to control.
(docm)

Figure S8 **Modulation of liver myofibroblats PCA.** Clotting time (CT) assayed by ROTEM after recalcification, with added tissue factor (ExTem 20 µL), of citrated whole blood (300 µl) in presence or not of liver myofibroblasts suspended in human albumin 5% with or without heparin (10 UI/ml) (Hepar). A combination of anticoagulant drugs was obtained when bivalirudin (Biva) was extemporaneously added to blood in contact with cells suspended in heparin *f* as compared to control.
(docm)

Figure S9 **HALPCs PCA and TF blocking antibody** Clotting time (CT) assayed by ROTEM after recalcification, with added tissue factor (TF) (ExTem 20 µL) of citrated whole blood (300 µl) in presence or not of cells suspended in human albumin 5% after the incubation of cells with TF antibody (TF+) or not (TF-). Hepatocytes (white), human adult liver progenitor cells (hALPCs) (black), control (albumin) (grey). * as compared to TF- for hALPCs $ as compared to TF- for hepatocytes *f* as compared to control.
(docm)

Figure S10 **Anti-Xa activity in plasma.** After a 30-min incubation of cells suspended in albumin with or without heparin (Hepar) (10 UI/ml, 50 UI/ml, and 100 UI/ml) in blood, anti-Xa activity (UI/ml) was measured in plasma obtained after blood centrifugation Human adult liver progenitor cells (hALPCs) (Black), Hepatocytes (Hep) (White), Control (Grey).
(docm)
