# Supplementary material for: Bivalirudin in Combination with Heparin to Control Mesenchymal Cell Procoagulant Activity
Source: PLoS One. 2012 Aug 10;7(8):e42819. doi: 10.1371/journal.pone.0042819 (PMC3416788; doi:10.1371/journal.pone.0042819)
Supplement: Figure S1 — Supernatant of hALPCs PCA. Clotting time (CT) essayed by ROTEM after recalcification, with added Tissue Factor (ExTem 20 µL), of citrated whole blood (300 µl) in presence of supernatant of hALPCs culture. No coagulation is induced if absence of recalcification. (docm) [file pone.0042819.s001.docm]

Figure S1-Supernatant of hALPCs PCA

Clotting time (CT) essayed by ROTEM after recalcification, with added Tissue Factor (ExTem 20μL), of citrated whole blood (300 µl) in presence of supernatant of hALPCs culture. No coagulation is induced if absence of recalcification.
