# Supplementary material for: Bivalirudin in Combination with Heparin to Control Mesenchymal Cell Procoagulant Activity
Source: PLoS One. 2012 Aug 10;7(8):e42819. doi: 10.1371/journal.pone.0042819 (PMC3416788; doi:10.1371/journal.pone.0042819)
Supplement: Figure S2 — Modulation of hALPCs PCA by heparin. A) Clotting time (CT) assayed by ROTEM after recalcification, with added tissue factor (ExTem 20 µL) of citrated whole blood (300 µl) in presence or not of human adult liver progenitor cells (hALPCs) (Black) suspended in human albumin 5% and with or without heparin (Hepar) at several concentrations (Hepar-10 UI/ml, Hepar 5×−50 UI/ml, and Hepar 10×−100 UI/ml) or not Control (albumin) (grey) f as compared to control. B) Clotting time (CT) assayed by ROTEM after recalcification, with added tissue factor (ExTem 20 µL) of citrated whole blood (300 µl) in presence or not of human adult liver progenitor cells (hALPCs) (Black) suspended in human albumin 5% and with or without fondaparinux (Fond) and enoxaparin (Eno) at normal concentrations or increased five times (5×) the normal concentration Control (albumin) (grey) f as compared to control Fond vs. Fond 5×, non-significant Eno vs. Eno 5×, non-significant. (docm) [file pone.0042819.s002.docm]

Figure S2-Modulation of hALPCs PCA by heparin

A) Clotting time (CT) assayed by ROTEM after recalcification, with added tissue factor (ExTem 20μL) of citrated whole blood (300 µl) in presence or not of human adult liver progenitor cells (hALPCs) (Black) suspended in human albumin 5% and with or without heparin (Hepar) at several concentrations (Hepar-10UI/ml, Hepar 5x-50UI/ml, and Hepar10x-100UI/ml) or not

Control (albumin) (grey)

*f* as compared to control

B) Clotting time (CT) assayed by ROTEM after recalcification, with added tissue factor (ExTem 20μL) of citrated whole blood (300 µl) in presence or not of human adult liver progenitor cells (hALPCs) (Black) suspended in human albumin 5% and with or without fondaparinux (Fond) and enoxaparin (Eno) at normal concentrations or increased five times (5x) the normal concentration

Control (albumin) (grey)

*f* as compared to control

Fond *vs.* Fond 5x, non-significant

Eno *vs.* Eno 5x, non-significant
