# Supplementary material for: Bivalirudin in Combination with Heparin to Control Mesenchymal Cell Procoagulant Activity
Source: PLoS One. 2012 Aug 10;7(8):e42819. doi: 10.1371/journal.pone.0042819 (PMC3416788; doi:10.1371/journal.pone.0042819)
Supplement: Figure S3 — Modulation of hALPCs PCA by hirudin. Clotting time (CT) assayed by ROTEM after recalcification, with added tissue factor (ExTem 20 µL) of citrated whole blood (300 µl) in presence or not of human adult liver progenitor cells (hALPCs) suspended in human albumin 5%. Increased concentrations of hirudin (Hir) at two (Hir 2x) or five times the normal levels (Hir 5x)) was extemporaneously added to blood. hALPCs (black), Control (albumin) (grey). * as compared to hALPCs f as compared to control hALPCs Hir vs. hALPCs Hir 2x, n.s. (docm). [file pone.0042819.s003.docm]

Figure S3- Modulation of hALPCs PCA by hirudin

Clotting time (CT) assayed by ROTEM after recalcification, with added tissue factor (ExTem 20μL) of citrated whole blood (300 µl) in presence or not of human adult liver progenitor cells (hALPCs) suspended in human albumin 5%. Increased concentrations of hirudin (Hir) at two (Hir 2x) or five times the normal levels (Hir 5x)) was extemporaneously added to blood.

hALPCs (black), Control (albumin) (grey)

* as compared to hALPCs

*f* as compared to control

hALPCs Hir *vs.* hALPCs Hir 2x, n.s.
