# Supplementary material for: Bivalirudin in Combination with Heparin to Control Mesenchymal Cell Procoagulant Activity
Source: PLoS One. 2012 Aug 10;7(8):e42819. doi: 10.1371/journal.pone.0042819 (PMC3416788; doi:10.1371/journal.pone.0042819)
Supplement: Figure S4 — Modulation of hALPCs PCA by bivalirudin. Clotting time (CT) assayed by ROTEM after recalcification, with added tissue factor (ExTem 20 µL) of citrated whole blood (300 µl) in presence or not of human adult liver progenitor cells (hALPCs) suspended in human albumin 5%. Increased concentrations of bivalirudin (Biva) two times the normal level (Biva 2x) was extemporaneously added to blood. hALPCs (black), Control (albumin) (grey). * as compared to hALPCs f as compared to control. (docm) [file pone.0042819.s004.docm]

Figure S4-Modulation of hALPCs PCA by bivalirudin

Clotting time (CT) assayed by ROTEM after recalcification, with added tissue factor (ExTem 20μL) of citrated whole blood (300 µl) in presence or not of human adult liver progenitor cells (hALPCs) suspended in human albumin 5%. Increased concentrations of bivalirudin (Biva) two times the normal level (Biva 2x) was extemporaneously added to blood.

hALPCs (black), Control (albumin) (grey)

* as compared to hALPCs

*f* as compared to control
