# Supplementary material for: Bivalirudin in Combination with Heparin to Control Mesenchymal Cell Procoagulant Activity
Source: PLoS One. 2012 Aug 10;7(8):e42819. doi: 10.1371/journal.pone.0042819 (PMC3416788; doi:10.1371/journal.pone.0042819)
Supplement: Figure S5 — Modulation of hALPCs by antithrombin activators in combination. Clotting time (CT) assayed by ROTEM after recalcification, with added tissue factor (ExTem 20 µL) of citrated whole blood (300 µl) in presence or not of human adult liver progenitor cells (hALPCs) suspended in human albumin 5% and with or without heparin (Hepar). Enoxaparin (Eno) or fondaparinux (Fond) was extemporaneously added to blood with cells suspended in heparin or not hALPCs (black), Control (albumin) (grey) f as compared to control. [file pone.0042819.s005.docm]

Figure S5-Modulation of hALPCs by antithrombin activators in combination

Clotting time (CT) assayed by ROTEM after recalcification, with added tissue factor (ExTem 20μL) of citrated whole blood (300 µl) in presence or not of human adult liver progenitor cells (hALPCs) suspended in human albumin 5% and with or without heparin (Hepar). Enoxaparin (Eno) or fondaparinux (Fond) was extemporaneously added to blood with cells suspended in heparin or not

hALPCs (black), Control (albumin) (grey)

*f* as compared to control
