# Supplementary material for: Bivalirudin in Combination with Heparin to Control Mesenchymal Cell Procoagulant Activity
Source: PLoS One. 2012 Aug 10;7(8):e42819. doi: 10.1371/journal.pone.0042819 (PMC3416788; doi:10.1371/journal.pone.0042819)
Supplement: Figure S6 — Modulation of hALPCs by direct inhibition of factor X in combination or not with bivalirudin. Clotting time (CT) assayed by ROTEM after recalcification, with added tissue factor (ExTem 20 µL) of citrated whole blood (300 µl) in presence or not of human adult liver progenitor cells (hALPCs) suspended in human albumin 5% with rivaroxaban. Combination of anticoagulant drugs was obtained when bivalirudin (Biva) was extemporaneously added to blood. hALPCs (black), Control (albumin) (grey). * as compared to hALPCs f as compared to control $ as compared to bivalirudin. (docm) [file pone.0042819.s006.docm]

Figure S6-Modulation of hALPCs by direct inhibition of factor X in combination or not with bivalirudin

Clotting time (CT) assayed by ROTEM after recalcification, with added tissue factor (ExTem 20μL) of citrated whole blood (300 µl) in presence or not of human adult liver progenitor cells (hALPCs) suspended in human albumin 5% with rivaroxaban. Combination of anticoagulant drugs was obtained when bivalirudin (Biva) was extemporaneously added to blood.

hALPCs (black), Control (albumin) (grey)

* as compared to hALPCs
*f* as compared to control

$ as compared to bivalirudin
