# Supplementary material for: Bivalirudin in Combination with Heparin to Control Mesenchymal Cell Procoagulant Activity
Source: PLoS One. 2012 Aug 10;7(8):e42819. doi: 10.1371/journal.pone.0042819 (PMC3416788; doi:10.1371/journal.pone.0042819)
Supplement: Figure S7 — Modulation of mesenchymal cells PCA by heparin. Clotting time (CT) assayed by ROTEM after recalcification, with added tissue factor (ExTem 20 µL) of citrated whole blood (300 µl) with human adult liver progenitor cells (hALPCs), hepatocytes, skin fibroblasts, bone marrow mesenchymal stem cells (BMMSC), bone marrow haematopoietic stem cells (BMHSC), or liver myofibroblasts suspended in human albumin 5% with or without heparin (10 UI/ml) (Hepar) f as compared to control. (docm) [file pone.0042819.s007.docm]

Figure S7-Modulation of mesenchymal cells PCA by heparin

Clotting time (CT) assayed by ROTEM after recalcification, with added tissue factor (ExTem 20μL) of citrated whole blood (300 µl) with human adult liver progenitor cells (hALPCs), hepatocytes, skin fibroblasts, bone marrow mesenchymal stem cells (BMMSC), bone marrow haematopoietic stem cells (BMHSC), or liver myofibroblasts suspended in human albumin 5% with or without heparin (10 UI/ml) (Hepar)

*f* as compared to control
