# Supplementary material for: Bivalirudin in Combination with Heparin to Control Mesenchymal Cell Procoagulant Activity
Source: PLoS One. 2012 Aug 10;7(8):e42819. doi: 10.1371/journal.pone.0042819 (PMC3416788; doi:10.1371/journal.pone.0042819)
Supplement: Figure S8 — Modulation of liver myofibroblats PCA. Clotting time (CT) assayed by ROTEM after recalcification, with added tissue factor (ExTem 20 µL), of citrated whole blood (300 µl) in presence or not of liver myofibroblasts suspended in human albumin 5% with or without heparin (10 UI/ml) (Hepar). A combination of anticoagulant drugs was obtained when bivalirudin (Biva) was extemporaneously added to blood in contact with cells suspended in heparin f as compared to control. (docm) [file pone.0042819.s008.docm]

Figure S8-Modulation of liver myofibroblats PCA

Clotting time (CT) assayed by ROTEM after recalcification, with added tissue factor (ExTem 20μL), of citrated whole blood (300 µl) in presence or not of liver myofibroblasts suspended in human albumin 5% with or without heparin (10 UI/ml) (Hepar). A combination of anticoagulant drugs was obtained when bivalirudin (Biva) was extemporaneously added to blood in contact with cells suspended in heparin

*f* as compared to control
