# Supplementary material for: Bivalirudin in Combination with Heparin to Control Mesenchymal Cell Procoagulant Activity
Source: PLoS One. 2012 Aug 10;7(8):e42819. doi: 10.1371/journal.pone.0042819 (PMC3416788; doi:10.1371/journal.pone.0042819)
Supplement: Figure S9 — HALPCs PCA and TF blocking antibody Clotting time (CT) assayed by ROTEM after recalcification, with added tissue factor (TF) (ExTem 20 µL) of citrated whole blood (300 µl) in presence or not of cells suspended in human albumin 5% after the incubation of cells with TF antibody (TF+) or not (TF-). Hepatocytes (white), human adult liver progenitor cells (hALPCs) (black), control (albumin) (grey). * as compared to TF- for hALPCs $ as compared to TF- for hepatocytes f as compared to control. (docm) [file pone.0042819.s009.docm]

Figure S9-hALPCs PCA and TF blocking antibody

Clotting time (CT) assayed by ROTEM after recalcification, with added tissue factor (TF) (ExTem 20μL) of citrated whole blood (300 µl) in presence or not of cells suspended in human albumin 5% after the incubation of cells with TF antibody (TF+) or not (TF-).

Hepatocytes (white), human adult liver progenitor cells (hALPCs) (black), control (albumin) (grey)

* as compared to TF- for hALPCs

$ as compared to TF- for hepatocytes

*f* as compared to control
