# Supplementary material for: Bivalirudin in Combination with Heparin to Control Mesenchymal Cell Procoagulant Activity
Source: PLoS One. 2012 Aug 10;7(8):e42819. doi: 10.1371/journal.pone.0042819 (PMC3416788; doi:10.1371/journal.pone.0042819)
Supplement: Figure S10 — Anti-Xa activity in plasma. After a 30-min incubation of cells suspended in albumin with or without heparin (Hepar) (10 UI/ml, 50 UI/ml, and 100 UI/ml) in blood, anti-Xa activity (UI/ml) was measured in plasma obtained after blood centrifugation Human adult liver progenitor cells (hALPCs) (Black), Hepatocytes (Hep) (White), Control (Grey). (docm) [file pone.0042819.s010.docm]

Figure S10--Anti-Xa activity in plasma

After a 30-min incubation of cells suspended in albumin with or without heparin (Hepar) (10 UI/ml, 50 UI/ml, and 100 UI/ml) in blood, anti-Xa activity (UI/ml) was measured in plasma obtained after blood centrifugation

Human adult liver progenitor cells (hALPCs) (Black), Hepatocytes (Hep) (White), Control (Grey)
